# Antioxidant, Antibacterial, and Antidiabetic Activities of *Aleuritopteris bicolor* From Nepal: A LC‐MS, In Vitro, and In Silico Investigations to Establish Its Potential as a Therapeutic Candidate

**DOI:** 10.1155/tswj/8851368

**Published:** 2026-01-14

**Authors:** Rekha Bhandari, Shailendra Kumar Sharma, Peru Kumari Bishwakarma, Sadikshya Sapkota, Ram Kishor Yadav, Harish Babu P. C., Sandesh Poudel, Sajan L. Shyaula, Khem Raj Joshi

**Affiliations:** ^1^ School of Health and Allied Sciences, Pokhara University, Pokhara, Kaski, Nepal, pu.edu.np; ^2^ Department of Analytical Services, Honeychem Pharma Analytical Services Private Limited, Bangalore, Karnataka, India; ^3^ Faculty of Science, National Academy of Science and Technology (NAST), Lalitpur, Nepal

**Keywords:** *Aleuritopteris bicolor*, antibacterial activity, antioxidant activity, in silico molecular docking, LC-MS analysis

## Abstract

*Aleuritopteris bicolor* (Family: Pteridaceae; Nepalese name: Raani Sinka) is an edible fern native to Nepal, India, China, Bangladesh, Sri Lanka, and Pakistan. Ethnomedicinal practitioners from various ethnic tribes in Nepal have traditionally prescribed it to heal wounds, diarrhea, dysentery, and gastritis. However, scientific evidence supporting these efficacies remains limited until January 2025. In the present study, we aimed to validate these traditional uses through chemical, in vitro, and in silico analyses of the plant′s hydroalcoholic extract. The 70% methanolic extract of the plant exhibited potent DPPH free radical scavenging activity with an IC_50_ of 20.54 ± 4.4 *μ*g/mL. The extract also demonstrated potent and dose‐dependent antibacterial activity against *Staphylococcus aureus* (zone of inhibition: 8–12 mm and minimum inhibitory concentration: 3125 *μ*g/mL), assessed using well diffusion and broth microdilution techniques. However, the plant extract was found to be a weak inhibitor of the enzyme alpha‐amylase. Phytochemical analysis using LC‐MS revealed the presence of chlorogenic acid, kumatakenin, quercetin 3‐*O*‐glucoside, and rhamnocitrin 3‐*O*‐glucoside; which, when docked against penicillin‐binding protein′s catalytic residues (SER 403, LYS 406, SER 462, ASN 464, and THR 600), showed binding energies ranging from −6.3 to −7.1 kcal/mol, suggesting prominent molecular interactions compared with meropenem antibiotics. Furthermore, kumatakenin satisfied Lipinski′s parameters for drug‐likeness, indicating its great potential as a drug candidate. The current study provides scientific evidence for the potent phytoconstituents with antioxidant and antibacterial potential of *A. bicolor* for the first time, highlighting its potential for topical application in treating bacterial infectious wounds.

## 1. Introduction

Human civilization has utilized plants for their remedial properties in various ailments since the time immemorable [1]. Currently, about 60% of the world′s population, including 80% in developing countries, relies on herbal medicine for primary care due to its safety and compatibility [2]. Plant extracts are increasingly important in pharmaceuticals for their cost‐effectiveness, renewability, and rich bioactive compounds that interact with a wide range of biological targets [3]. They offer promising alternatives to synthetic drugs, aiding in the discovery of new medicines. The herbal medicine market exceeds 1 trillion dollars and grows over 8% annually, driven by consumer preference for natural products [4]. Research into pharmacologically valuable plants is vital for developing novel drugs and advancing healthcare [5].

Respiration is the central metabolic pathway of all living cells serving to produce ATPs, the energy currency of the cells. With a theoretical energy conversion efficiency of 38%, aerobic respiration is the most efficient respiratory pathway identified [5]. Despite the necessity of the respiratory process for life, respiration is known to yield several by‐products of which free radicals are identified as notorious to cells. These free radicals exert oxidative damage to cell organelles and biomolecules [6]. The oxidative damages exerted by these free radicals have been traced to be the chief contributors in the pathogenesis of several diseased conditions, including cancer, diabetes, atherosclerosis, ulcer, hypertension, neurodegeneration, thyroid disorder, and many more [5, 7, 8]. In this regard, the use of scavengers of free radicals points towards an effective mechanism of therapy for diseases concerned with oxidative damages. Plant‐based antioxidants are among the most researched free radical scavengers and have demonstrated their roles in numerous scientific studies [7, 9, 10].

Globally, the emergence and spread of antibiotic resistance, along with the evolution of new bacterial strains, pose significant concerns for human health [11]. The World Health Organization (WHO) identifies antimicrobial resistance (AMR) as one of the most critical threats to health and development, accounting for approximately 1.27 million deaths in 2019 and contributing to a total of 4.95 million deaths [12]. The misuse and abuse of antimicrobials have significantly increased the prevalence of antibiotic resistance, especially among pathogens such as *Escherichia coli*, *Klebsiella pneumoniae*, *Staphylococcus aureus*, *Pseudomonas aeruginosa*, *Acinetobacter baumannii*, and *Streptococcus pneumoniae*. The discovery and development of new antibiotics have not kept pace with the rate of emerging resistance. Therefore, it is imperative for researchers in drug discovery to prioritize the identification of new, effective, and safer alternatives to combat the threat of AMR. Medicinal plants rich in alkaloids, flavonoids, and phenolic compounds have demonstrated promising and broad‐spectrum antibacterial activities in scientific investigations [13].

Despite the recognized medicinal value of plants, there is limited chemical and pharmacological data available for many traditional medicinal species, including *Aleuritopteris bicolor*. The rise of antibiotic resistance and oxidative stress highlights the need for safer, effective alternatives, prompting the exploration of plant‐based compounds with antimicrobial and other therapeutic properties [1]. Additionally, understanding the chemical constituents and biological activities of *A. bicolor* can support its ethnomedicinal uses and promote its potential application in modern medicine.

*A. bicolor* (Roxb.) Fraser‐Jenk (synonym: *Cheilanthes bicolor*) is an edible fern belonging to the Pteridaceae family. It is commonly found in moist environments, including rocky areas exposed to sunlight and shady forests [14, 15]. It is locally known as Kali Sinki, Raani Sinka, and Dankernu in Nepal [16]. The plant is native to Nepal, India, China, Bangladesh, Sri Lanka, and Pakistan. The Tamang and Chepang communities in various districts of Nepal use this plant to treat sinusitis, fever, and cuts. Additionally, the juice extracted from the whole plant is used to manage diarrhea, dysentery, gastritis, and bites from black spiders and scorpions [17].

Studies investigating the scientific basis of these claims have shown that the plant contains secondary metabolites such as polyphenols, glycosides, coumarins, and flavonoids [18]. In our previous research, we found that hydroethanolic extracts of the plant are rich in polyphenols, flavonoids, and saponins and exhibit strong free radical scavenging activity. Additionally, we observed that these extracts have moderate alpha‐amylase inhibitory effects and notable analgesic and anti‐inflammatory properties [14, 15]. However, to our knowledge, information regarding the chemical constituents of *A. bicolor* remains limited.

This study is aimed at filling the existing research gaps by characterizing the chemical constituents of *A. bicolor* and evaluating its biological activities, specifically its antioxidant, antibacterial, and antidiabetic effects. Validating its ethnomedicinal applications through scientific methods can support its development as a natural therapeutic agent. Moreover, employing in silico techniques can provide insights into its mechanism of action underlying *A. bicolor* bioactivity, which could enhance its topical application for superficial bacterial infections.

## 2. Materials and Methods

### 2.1. Chemicals and Bacterial Strain

Ascorbic acid (Merck, India), methanol (Thermo Fisher Scientific, India), Mueller Hinton agar, amikacin disc, meropenem disc, and quercetin dihydrate (HiMedia, India); 2,2‐diphenyl‐1‐picrylhydrazyl (DPPH); and *α*‐amylase (SRL, India) were used for the study. Bacterial strains, including *S. aureus* (ATCC 11238)*, K. pneumoniae* (ATCC 70065), *P. aeruginosa (*ATCC 9027), and *E. coli* (ATCC 11386), were obtained from Manipal Teaching Hospital, Pokhara, Nepal.

### 2.2. Extraction Procedure

Aerial parts of *A. bicolor* were collected from Pokhara‐30, Nepal. The scientific name of the plant specimen was confirmed at National Herbarium and Plant Laboratories, Godawari‐3, Lalitpur, Nepal. The aerial parts of *A. bicolor* were shade‐dried at room temperature for 1 week to remove moisture content. The plant material was then cut into smaller sizes using a plant cutter. Extraction was carried out using 70% MeOH with a sample‐to‐solvent ratio of 1:10 (*w*/*v*) at 55°C for 5 h, followed by room temperature (approximately 24°C) for 67 h, as described in our previous method [19]. The liquid crude extract was filtered using a thick cotton bed and then used for qualitative phytochemical analysis. Meanwhile, the liquid extract was further subjected to rotary evaporator (Biobase RE‐2000B, Germany) maintained at reduced pressure and temperature of 55°C, followed by a vacuum desiccator to complete drying process for quantitative analysis. After complete drying, the extracts were weighted to estimate the extraction yield, which was followed by storage at 4°C.

Extraction yield (*%*) = (weight of dry extract/weight plant used for extraction) × 100.

### 2.3. Qualitative Phytochemical Analysis

Phytochemical profile of the plant was established adopting previous methods [20–22]. Plant metabolites such as alkaloids, phenols, flavonoids, tannins, carbohydrates, saponins, terpenoids, and proteins in *A. bicolor* extracts were tested.

### 2.4. Quantitative Phytochemical Analysis

#### 2.4.1. Total Phenolic Content (TPC)

Total phenol in *A. bicolor* extract was quantified spectrophotometrically by implementing the Folin–Ciocalteus reagent method [6]. For this, 100 *μ*L of extract (1000 *μ*g/mL in methanol) and 0.5 mL Folin–Ciocalteu phenol reagent (2 N) were mixed thoroughly and diluted with 6 mL distilled water. After allowing to stand for 5 min, 1.5 mL of sodium carbonate (7.5%) was added followed by dilution with 1.9 mL of distilled water. The reaction mixture was then incubated for 2 h in the dark. Standard solutions of gallic acid (31.2–500 *μ*g/mL) were treated similarly to the extract. At the end of 2 h, the absorbance (*λ*_max_ = 750 nm) of each of the samples were measured using a single‐beam UV‐VIS spectrophotometer (Agilent Cary 60, Malaysia) against appropriate blank solutions. The mean absorbance of triplicates per sample was recorded, and TPC was expressed as milligram of gallic acid equivalents (mg of GAE)/g of extract.

#### 2.4.2. Total Flavonoid Content (TFC)

Quantification of total flavonoids in *A. bicolor* extract was done adopting a spectrometric method that involves formation of yellow coloration of flavonoids when treated with AlCl_3_ as previously described [6]. For this, equal volumes of AlCl_3_ (2% in methanol) and the plant extract (100 *μ*g/mL) were mixed and incubated at room temperature for 10 min. Standard solutions of quercetin (1.25–100 *μ*g/mL in methanol) were used as reference and were treated similarly to the extract. Absorbance was recorded with a single‐beam UV‐VIS spectrophotometer at 415 nm against a blank containing the extract with methanol. The mean absorbance of triplicates per sample was recorded and expressed as milligrams of quercetin equivalent (mg of QE)/g of extract.

#### 2.4.3. Total Carbohydrate Content (TCC)

TCCs were estimated as per our previous methods [19]. Briefly, 1 mL of plant extract (250 *μ*g/mL) was mixed with 0.5 mL phenol solution and 2.5 mL H_2_SO_4_. After incubating for 30 min at room temperature, the absorbance was measured at 490 nm against the blank containing distilled water instead of extract. D‐glucose was employed as the standard carbohydrate, and concentrations ranging from 12.5 to 200 *μ*g/mL were used to plot the calibration curve. The TCC was calculated as milligrams of D‐glucose equivalent (GE)/g of *A. bicolor* extract.

### 2.5. Thin‐Layer Chromatography (TLC) Profiling

TLC profiling was conducted according to our previous method [19]. Approximately 1 mg/mL of particle‐free plant extract was used for this purpose. With the aid of a microcapillary tube, a band of the extract was drawn at the base of precoated silica gel 60 F_254_ plates. The plates were developed in a saturated glass beaker containing a mixture of solvents as mobile phases (chloroform, methanol, and water = 7 : 3 : 0.5). The developed plates were dried using a hot air blower. Visualization was aided with UV light at 254 and 365 nm, followed by the derivatization with 10% *w*/*v* FeCl_3_ spray/dry, 10% *v*/*v* H_2_SO_4_ spray/heat, and dipping in DPPH solution.

### 2.6. Liquid Chromatography‐Mass Spectrometry (LC‐MS) Analysis

The 70% MeOH extract of *A. bicolor* was analyzed using a LC‐ electrospray ionization (ESI)/MS system [1, 21]. Reverse phase chromatographic system was adopted equipping a C‐18 column (Waters XBridge 50 × 4.6 mm, 3.5 *μ*) maintained at 35°C. A linear gradient of mobile phase was programmed optimally mixing 0.1% formic acid in water (*A*) and 100% acetonitrile (*B*) maintaining a flow at the rate of 1.2 mL/min such that (Time − *%* *A*/*%* *B*): 0 min‐85/15, 6 min‐25/75, and 11–15 min‐85/15. The extract concentration was 1 mg/mL, and 5 *μ*L was injected for the analysis. Single quadrupole mass analyzer was used as the detection system for MS. ESI source was operated in both positive (ESI+) and negative (ESI−) ion modes and the scanning range of *m*/*z* 0–1000. The MS source parameters were set as follows: ESI capillary voltage, 3.03 KV; cone voltage, 13 V; source temperature, 118°C; desolvation temperature, 246°C; and gas flow rate, desolvation (500 L/h) and cone (50 L/h).

### 2.7. In Vitro Biological Activity

#### 2.7.1. Antioxidant Activity

Antioxidant activity of the *A. bicolor* extract was evaluated using DPPH free radical scavenging protocol [20]. For this, equal volumes of plant extracts of varying concentrations were treated with freshly prepared DPPH methanolic solution (100 *μ*M) in a titer plate. The mixtures were incubated for 30 min in dark; then, their absorbances were measured at 517 nm against the DPPH control (equal volumes of DPPH solution and methanol) and blank (methanol). Standard solutions of ascorbic acid (0.6125–10 *μ*g/mL) were used as a positive control. Experiments were done in triplicate, and their mean absorbance was used to calculate the percentage of DPPH^•^ scavenging activity.

*%*DPPH radical scavenging = [(*A*_0_ − *A*_1_)/*A*_0_] × 100.

Here, *A*_0_ represents the absorbance of the DPPH control and *A*_1_ represents the absorbance of the sample or positive control. The antioxidant potency of each extract sample and ascorbic acid was expressed as an IC_50_ value (mean ± standard deviation). The linear graph showing the percentage of DPPH scavenging activity versus extract or ascorbic acid concentrations was utilized to calculate the IC_50_ value [14].

#### 2.7.2. Antibacterial Activity

The antibacterial activity of *A. bicolor* extracts was evaluated against gram‐positive (*S. aureus*), and gram‐negative bacteria (*K. pneumonia*, *P. aeruginosa*, and *E. coli*), employing well diffusion and broth microdilution methods as per our previous method [1, 19].

##### 2.7.2.1. Well Diffusion Assay

Muller–Hinton agar was autoclaved and poured aseptically into sterile Petri plates. After solidification of the culture media, standard bacterial inoculums (0.5 McFarland) were swabbed over the entire surface of the MHA plates. Using a sterile tip (6 mm), five wells were created in each plate. A total of 20 *μ*L of molten MHA was dropped to seal the base of the wells. Afterwards, 100 *μ*L of *A. bicolor* extract at different concentrations (25, 50, and 100 mg/mL in sterile water) was added to the respective wells, and the plates were placed in an incubator for 48 h at 37°C. A negative control (sterile water) and a positive control (amikacin 30 *μ*g disc and meropenem 10 *μ*g disc) were used. After 48 h, the clear zone of inhibition (ZOI) around the wells was measured in millimeter (mm).

##### 2.7.2.2. Broth Microdilution Assay

The sensitive bacterial strain against the *A. bicolor* extract was further analyzed for their potency (minimum inhibitory concentration (MIC)) using broth microdilution method [1, 23]. Briefly, 100 *μ*L of different extract concentrations (3.90625–5000 *μ*g/mL) and meropenem (0.3906–50 *μ*g/mL) were treated with 100 *μ*L of *S. aureus* suspension (0.5 McFarland standard) on a 96‐well titer plate and then incubated for 24 h at 37°C. Additionally, resazurin dye was added in each well to monitor the growth of *S. aureus*. The color change from blue to pink indicated bacterial growth. The minimum concentration showing no growth on visual observation (blue color) was considered as the MIC against the negative control containing Muller–Hinton broth instead of extract or antibiotics.

#### 2.7.3. Antidiabetic Activity

The antidiabetic activity of *A. bicolor* extract was assessed by adopting *α*‐amylase inhibitory method [6]. A 1 mL of *A. bicolor* extract at concentrations (6.25–1000 *μ*g/mL) was mixed with 5mL porcine pancreatic *α*‐amylase (pH 6.9, 4 U/mL in 20 mM phosphate‐buffer saline at pH 6.9) in a test tube. After a 5‐min incubation at 25°C, 2 mL of 0.5% starch solution was added and then incubated at 25°C for an additional 3 min. Following this, 2mL mixture of test tube was added to a separate test tube containing 1 mL of 3,5‐dinitrosalicylic acid reagent solution and placed in water bath maintained at 85°C. After 15 min, the mixture was diluted with 3 mL of distilled water, and absorbance was measured at 540 nm against control containing PBS instead of extract and blank containing alpha‐amylase solution. Acarbose was used as a positive control. The inhibitory activity was calculated as follows:

α−Amylase Inhibition %=Acontrol−Asample/Acontrol×100,

where *A* represents the absorbance of the sample or control.

### 2.8. In Silico Study

#### 2.8.1. Molecular Docking

The phytoconstituents of *A. bicolor* identified by LC‐MS analysis were chosen as ligands and docked against the penicillin‐binding protein, following our previous method [5]. The 3D structure of phytoconstituents and standard antibiotic meropenem was retrieved from the PubChem database in the SDF (structure data format) and then converted to PDB (Protein Data Bank) format using BIOVIA Discovery Studio Visualizer. The ligands were optimized by removing extra moieties and adding crucial polar hydrogen and Kollman charge and finally converted to the pdbqt files using AutoDock 1.5.6. software. Likewise, penicillin‐binding protein (PBP 2a) was selected as the target protein [5]. The 3D crystal structure of PBP 2a (PDB ID: 6H5O) was downloaded from the RSCB PDB server and subjected to purification by eliminating (water and heteroatoms) and joining (polar hydrogens and Kollman charge). The energy of the protein was minimized and finally converted to pdbqt format using AutoDock 1.5.6. software.

**Figure 1:**
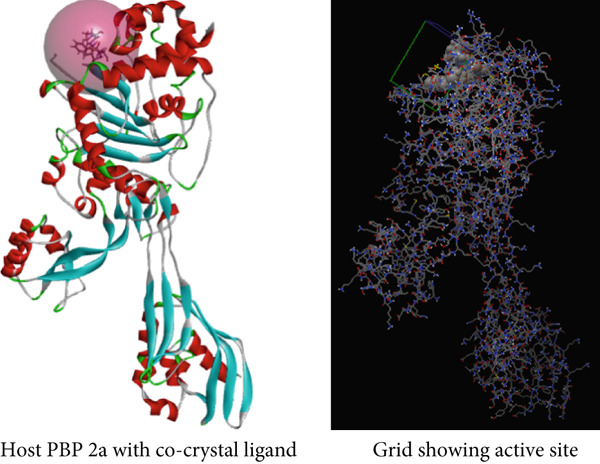
3D structure of the host protein (PBP 2a) and its active site amino acids represented by a grid box.

AutoDock Vina 1.5.7. was used for docking the ligands against PBP 2a in a 3D space defined by the grid box of 20 × 20 × 20 sizes with coordinates: *x* = 36.71, *y* = −0.95, and *z* = 24.47 with a spacing 0.375 Å, enclosing active site amino acid residues (Figure 1). After docking, BIOVIA Discovery Studio Visualizer 2020 was utilized to analyze the interactions between the docked protein and ligands. Redocking and superimposition techniques were applied to validate the docking protocol [24].

#### 2.8.2. Drug‐Likeness

Lipinski biopharmaceutical parameter was determined to predict the drug‐likeness of native phytoconstituents of *A. bicolor* using computation SwissADME software [1].

## 3. Results and Discussions

### 3.1. Extraction Yield

The choice of solvent system plays a crucial role in extraction to obtain the target bioactive compounds and their associated biological activities [1]. In this study, we aimed to extract polar bioactive compounds, including phenols, flavonoids, and terpenoids; therefore, 70% methanol (MeOH) was selected as the extraction solvent [25–27]. Additionally, a temperature of 55°C was maintained in the first 5 h of extraction to deactivate the microbes and achieve a higher extraction yield [22]. The application of heat during extraction was found to improve the extraction efficiency by thermal destruction of the plant cell wall, which enhances the solubility and release of intracellular plant constituents [28, 29]. In the current study, the 70% methanolic solvent yielded 27.95% extract from *A. bicolor*. Previously, KC et al. reported an extraction yield of 11.23% from the leaves of *A. bicolor*, using 70% ethanol as the solvent [15]. Similarly, Jha et al. observed an extraction yield of 15.48% using 80% ethanol for *A. bicolor* [30]. The higher extraction yield in this investigation may be attributed to the higher polarity of the extraction solvent and the application of heat during the process of extraction.

### 3.2. Qualitative Phytochemical Analysis

Phytochemical analysis of *A. bicolor* indicated the presence of phenols, tannins, flavonoids, saponins, steroids, terpenoids, carbohydrates, and protein, whereas alkaloids and anthraquinones were absent (Table 1). These findings were found to be consistent with several previous studies revealing the accumulation of a similar class of phytoconstituents in *A. bicolor* [15, 18, 31].

**Table 1:** Qualitative phytochemical analysis of *A. bicolor* extract.

**S. N.**	**Phytochemicals**	**Types of test**	***A. bicolor* extract**
1	Phenols	1. FeCl_3_ tests	+
2	Flavonoids	1. Alkaline reagent tests	+
		2. Lead acetate tests	+
3	Tannins	1. FeCl_3_ tests	+
		2. Lime water tests	+
		3. Gelatin tests	+
4	Saponins	1. Foam tests	+
5	Alkaloid	1. Mayer′s tests	−
		2. Dragendorff tests	−
6	Triterpenoid	1. Salkowski tests	+
7	Carbohydrate	1. Fehling′s tests	−
		2. Benedict′s tests	+
8	Protein	1. Ninhydrin tests	+
		2. Biuret tests	−

*Note:* (+) indicates presence; (–) indicates absence.

### 3.3. Quantitative Phytochemical Analysis

The TPC, TFC, and TCC were estimated using linear regression equations derived from calibration curves of standard compounds: gallic acid (*y* = 0.0014*x* − 0.0481, *R* ^2^ = 0.98), quercetin (*y* = 0.034*x* − 0.017, *R* ^2^ = 0.98), and D‐glucose (*y* = 0.0021*x* + 0.79, *R* ^2^ = 0.95). The analyses revealed that *A. bicolor* contains phenolics, flavonoids, and carbohydrates at levels of 227.93 ± 4.2 mg GAE/g, 260.13 ± 2.1 mg QE/g, and 156.11 ± 12.1 mg GE/g of dry extract, respectively. In a previous study, Tiwari et al. reported a TPC of approximately 90 mg of GAE and a TFC of 429 mg of quercetin equivalents (QEs) in a 60% ethanolic extract of *A. bicolor* [18]. Similarly, research on a 70% ethanolic extract reported a TPC of 28.13 ± 0.57 mg GAE and a TFC of 398.86 ± 6.94 mg QE from *A. bicolor* leaves [15]. Furthermore, Prabhat and colleagues reported a TPC of 20.98 ± 0.20 mg GAE per gram of dry extract and a TFC of 405.95 ± 0.28 mg QE per gram of dry extract in an 80% ethanolic extract of same plant species [31].

### 3.4. TLC Profiling

**Figure 2:**
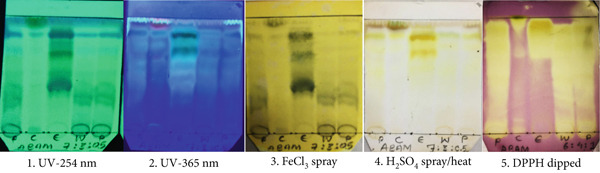
Thin layer chromatography (TLC) profiles of 70% MeOH extract of *A. bicolor* (P), and its chloroform (C), ethyl acetate (E), and water (W) fractions, using silica gel F_254_ as stationary phase and chloroform: methanol: water = 7 : 3 : 0.5 v/v as development solvent; observed under (1) UV‐254 nm, (2) UV‐365 nm, (3) using spray reagent 10% FeCl_3_ and dry, (4) using spray reagent 10% H_2_SO_4_ and heated, and (5) dipped in 500 *μ*M DPPH solution.

TLC is a simple and widely used technique for the rapid evaluation of phytoconstituents in plant extracts and herbal medicines [1, 5]. It employs several derivatizing reagents for the detection of specific classes of phytochemicals. For example, FeCl_3_, H_₂_SO_₄_, and DPPH solutions are sprayed for the identification of phenolics, flavonoids, and antioxidants, respectively [1, 5]. Figure 2 illustrates the TLC profiles of 70% MeOH extract of *A. bicolor.* Several bands were observed on TLC when visualized under UV light, which confirmed the presence of UV active phytoconstituents, such as phenols, flavonoids, and terpenoids. Specifically, the green, blue, and brown bands on the TLC of different fractions of 70% MeOH extract of *A. bicolor*, after spraying with 10% FeCl_3_ as a derivatizing agent, suggest the presence of phenolic compounds, such as catechol, galloyl, and phenol groups. Likewise, the yellow and reddish‐brown bands on the TLC, after H_2_SO_4_ spray/heat, indicates the presence of flavonoids and terpenoids, respectively [32]. Furthermore, a yellow band on violet background appeared after dipping the developed TLC plate into the DPPH solution, suggesting the presence of antioxidants in the extract of *A. bicolor* [22].

### 3.5. LC‐MS Analysis

**Figure 3:**
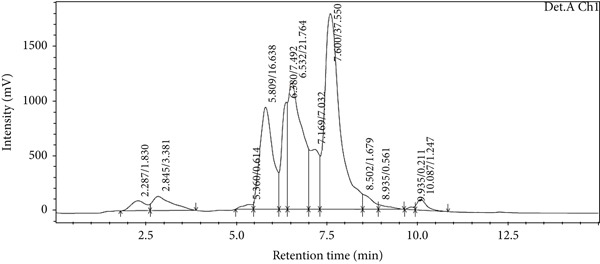
HPLC chromatogram (254 nm) of 70% MeOH extract of *A. bicolor.*

**Figure 4:**
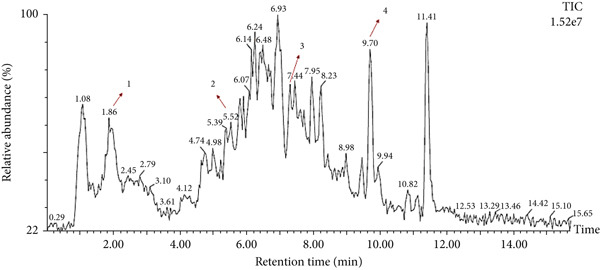
Liquid chromatography‐mass spectrometry (LC‐MS) acquired for 70% MeOH extract of *A. bicolor.*

**Figure 5:**
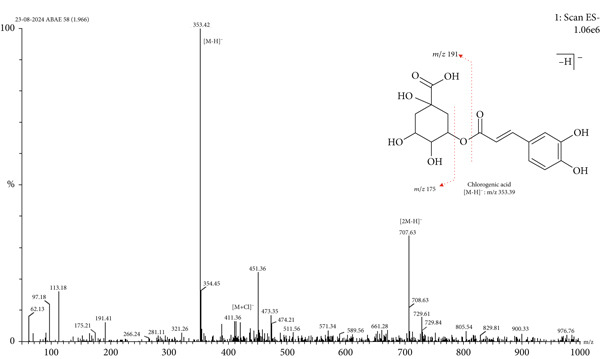
Mass spectra of Peak 1 (retention time of 1.96 min) indicating the deprotonated molecular ion at *m*/*z* 353.42 [*M* − *H*]^−^, adduct ion at *m*/*z* 389.37 [*M* + Cl]^−^, and dimer ion at *m*/*z* 707.63 [2 *M* − *H*]^-^ in negative mode ESI. Additionally, the fragment ion at *m*/*z* 191.18 indicates quinic acid formed after the loss of caffeoyl (C_9_H_6_O_3_; 162 Da) from the precursor ion *m*/*z* 353.39 [6, 37]. By comparing these spectral data with previously isolated compounds from same genus *Cheilanthes* [38], Peak 1 was identified as chlorogenic acid.

**Figure 6:**
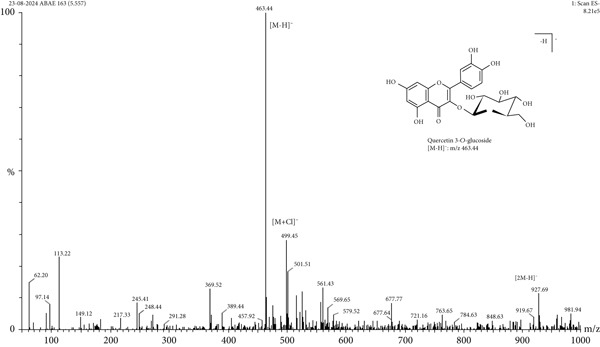
Mass spectra of Peak 2 (retention time of 5.5 min) indicating the deprotonated molecular ions at *m*/*z* 463.44 [*M* − *H*]^−^, adduct ion at *m*/*z* 499.45 [*M* + Cl]^−^, and dimer ion at *m*/*z* 927.69 [2 *M* − *H*]^−^ in negative mode ESI. By comparing these spectral data with previously isolated compounds from same genus *Cheilanthes* [39], Peak 2 was identified as quercetin 3‐*O*‐glucoside.

**Figure 7:**
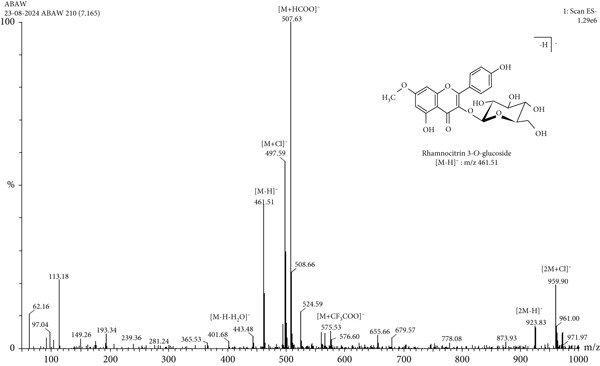
Mass spectra of Peak 3 (retention time of 7.165 min) indicating the deprotonated molecular ions at *m*/*z* 461.51 [*M* − *H*]^−^, adduct ion at *m*/*z* 497.59 [*M* + Cl]^−^, 507.63 [*M* + HCOO]^−^, 575.53 [*M* + CF_3_COO]^−^, and dimer ion at *m*/*z* 923.83 [2 *M* − *H*]^−^ and 959.90 [2 *M* + Cl]^-^ in negative mode ESI. By comparing these spectral data with previously isolated compounds from close species of genus *Cheilanthes* [36], Peak 3 was identified as rhamnocitrin 3‐*O*‐glucoside.

**Figure 8:**
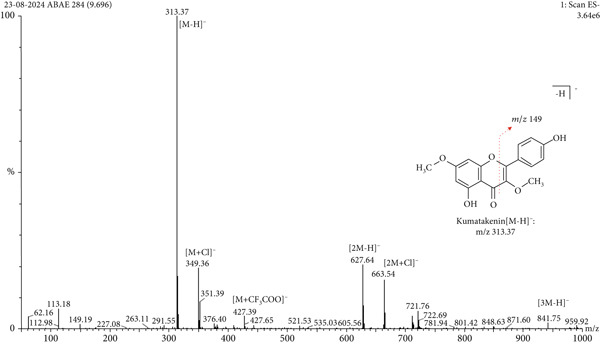
Mass spectra of Peak 4 (retention time of 7.165 min) indicating the deprotonated molecular ions at *m*/*z* 313.37 [*M* − *H*]^−^, adduct ion at *m*/*z* 349.36 [*M* + Cl]^−^, 427.39 [*M* + CF_3_COO]^−^, and dimer ion at *m*/*z* 627.64 [2 *M* − *H*]^−^ and 663.54 [2 *M* + Cl]^−^in negative mode ESI. Additionally, the fragment ion at *m*/*z* 149 was formed after the retro Diels–Alder cleavage from the precursor ion *m*/*z* 313.37 [40, 41]. By comparing these spectral data with previously isolated compounds from same genus *Cheilanthes* [36], Peak 4 was identified as kumatakenin.

**Figure 9:**
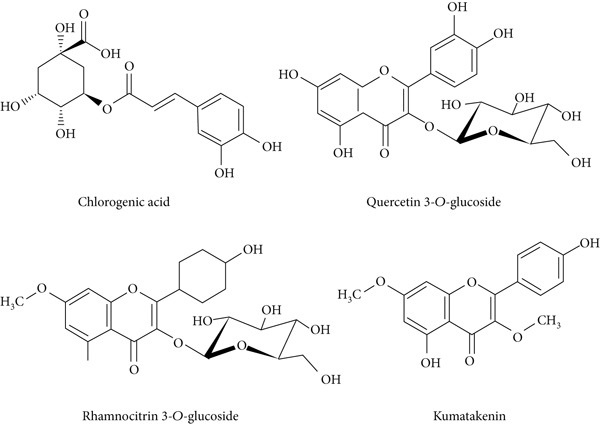
Compounds identified from 70% MeOH extract of *A. bicolor.*

LC‐MS is a sophisticated analytical technique used for comprehensive phytoconstituents analysis, renowned for its high sensitivity and specificity [14]. It is widely applied in fields such as pharmaceuticals, forensic toxicology, clinical research, and food and environmental testing [5]. This study focuses on the qualitative analysis of phytochemicals in *A. bicolor* extract using LC‐MS equipped with ESI, a gentle ionization method that results in minimal fragmentation [33]. The identification of phytoconstituents was based on their mass‐to‐charge ratios (*m*/*z*). In ESI+, ESI typically produces protonated molecules [*M* + *H*]^+^, whereas in ESI−, deprotonated molecules [*M* − *H*]^−^ are generated [1, 5]. Moreover, depending on the mobile phase composition, ESI can produce adduct ions such as cationized molecules [M + Na]^+^, [M + NH4]^+^, [M + K]^+^, [2 M + H]^+^, and [2 M + Na]^+^ in ESI+ mode, and anionized molecules [M + Cl]^−^, [2 M − H]^−^, [M + HCOO]^−^, and [M + CH_3_COO]^−^ in ESI− mode [33–35]. For this analysis, the ESI− was chosen due to its lower background noise and higher ionization efficiency [1, 5]. Four polyphenolic phytoconstituents, including chlorogenic acid, quercetin 3‐*O*‐glucoside, rhamnocitrin 3‐*O*‐glucoside, and kumatakenin were identified by analyzing their spectral data, including deprotonated ions, adduct ions, dimer ions, and by comparing these with existing literature of *Aleuritopteris* closer species [36]. The high‐performance liquid chromatogram and mass spectrum are shown in Figures 3, 4, 5, 6, 7, 8, and 9 and Table 2.

**Table 2:** Phytoconstituents identified by liquid chromatography‐mass spectrometry in 70% MeOH extract of *A. bicolor* from Nepal.

**Peak no.**	**RT (min)**	**Identification**	**Molecular formula**	**Molecular weight** **m**/**z**	**Experimental** **m**/**z** **[** **M** − **H** **]**	**Adduct ion** **m**/**z**	**Fragments** **m**/**z**
1.	1.96	Chlorogenic acid	C_16_H_18_O_9_	354.31	353.42	411.36, 707.63	191.41, 175.21
2.	5.55	Quercetin 3‐*O*‐glucoside	C_21_H_20_O_12_	464.38	463.44	499.45, 927.69	—
3.	7.16	Rhamnocitrin 3‐*O*‐glucoside	C_22_H_22_O_11_	462.4	461.51	497.59, 507.6, 575.53, 923.83, 959.9	—
4.	9.69	Kumatakenin	C_17_H_14_O_6_	314.29	313.37	349.36, 427.39, 627.64, 663.54,	149.19

### 3.6. Antioxidant Activity

**Figure 10:**
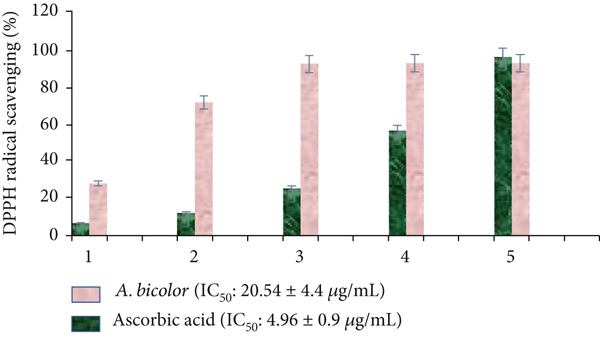
Dose‐dependent free radical scavenging activity of *A. bicolor* extract and standard ascorbic acid.

**Figure 11:**
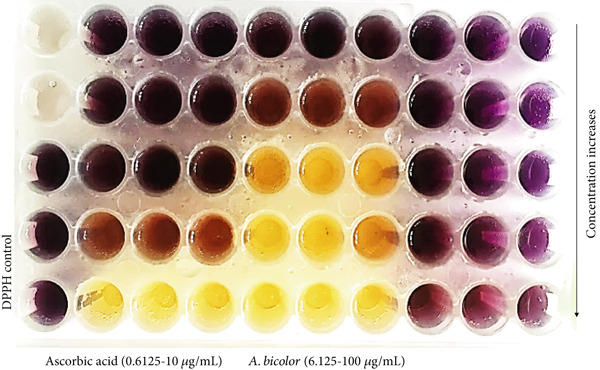
Antioxidant activity evaluation of *A. bicolor* extract and standard ascorbic acid by DPPH free radical scavenging assay. Violet color represents the DPPH free radical (control), whereas yellow color at the higher concentration of *A. bicolor* extract/ascorbic acid indicated complete scavenging of free radicals.

Antioxidant activity of *A. bicolor* extract was assessed by using a reliable protocol, DPPH free radical scavenging assay [42]. Antioxidants donate hydrogen atoms to scavenge the 2,2‐diphenyl‐1‐picrylhydrazyl (free radical: violet/purple color), converting it to 2,2‐diphenyl‐1‐picrylhydrazine (nonradical: yellow color) [5]. This process is monitored by the absorbance reading at 517 nm (Figures 10 and 11). In this assay, *A. bicolor* extract scavenges the DPPH free radical in a dose dependent manner, with an IC_50_ value of 20.54 ± 4.4 *μ*g/mL, compared with standard ascorbic acid which has an IC_50_ value of 4.96 ± 0.04 *μ*g/mL. A prior study on the same species, *A. bicolor* leaves, reported the similar DPPH scavenging activity of its hydroethanolic extract, with an IC_50_ value ranging from 3.23 to 39.87 *μ*g/mL [14, 15]. The promising antioxidant properties of *A. bicolor* extract are likely due to its native phenolic and flavonoid compounds, including chlorogenic acid, quercetin 3‐*O*‐glucoside, rhamnocitrin 3‐*O*‐glucoside, and kumatakenin [42, 43]. Previously, Joshi et al. [25] and Lamichhane et al. [36] have reported the promising DPPH scavenging activity of chlorogenic acid, quercetin 3‐*O*‐glucoside, rhamnocitrin 3‐*O*‐glucoside, and kumatakenin, being IC_50_ value of 178.2, 54.6, 200.23, and 178.57 *μ*M, respectively. These phytochemicals possess strong antioxidant activity that protects human tissue against oxidative stress and damage from free radicals and reactive oxygen species [10]; therefore, it suggests the immense potential of the *A. bicolor* extract for entering to the pipelines of antioxidant drug discovery and development.

### 3.7. Antibacterial Activity

**Figure 12:**
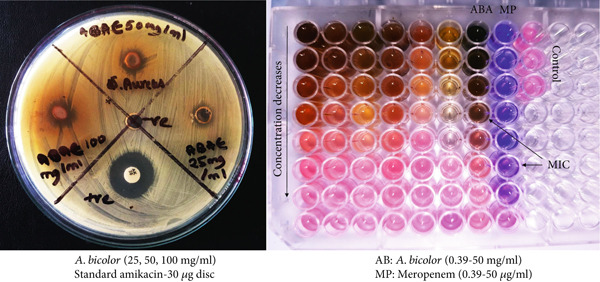
Photographs showing the antibacterial zone of inhibition (ZOI) and minimum inhibitory concentration (MIC) of *A. bicolor* and standard amikacin against *S. aureus.* The clear diameter around each well of the Petri plate indicated the ZOI in mm. Likewise, MIC indicated the lowest concentration of test extract/standard antibiotic showing no bacterial growth on visualization (blue color) against the negative control (pink) confirming the bacterial growth.

The worldwide spread and dissemination of antibiotic resistance, along with the emergence of new bacterial strains, present a substantial threat to public health [11]. Consequently, the effective management of infectious diseases requires thorough research and the development of innovative therapeutics derived from potent phytochemical sources. Natural products have provided a diverse array of antibacterial lead compounds that serve as effective agents in combating infections [5]. The in vitro antibacterial assay showed a dose‐dependent antibacterial activity of *A. bicolor* extract against *S. aureus* (Table 3 and Figure 12). The disc diffusion method indicated a clear ZOI in the range of 8–12 mm. Additionally, the broth dilution assay demonstrated the antibacterial potency of *A. bicolor* extracts in terms of MIC value of 3125 *μ*g/mL, compared with standard meropenem (MIC: 1.56 *μ*g/mL). Similarly, a previous study on a 60% ethanolic extract of *A. bicolor* reported significant inhibitory activity against *S. pneumoniae* (ZOI: 18 mm at 200 *μ*g/mL), while showing no activity against *S. aureus* and *Enterococcus* species [18]. This contradictory claim may result from the emergence of resistance in the *S. aureus* strain or from the minimal presence of antibacterial phytoconstituents in the extract [1, 5]. The effective antibacterial activity of *A. bicolor*, in the current investigation, characterized by a high ZOI and low MIC values, may be attributed to the synergistic effects of the cognate phenolic compounds such as chlorogenic acid, quercetin 3‐*O*‐glucoside, rhamnocitrin 3‐*O*‐glucoside, and kumatakenin [44–50].

**Table 3:** Antibacterial activity of *A. bicolor* extract using agar well diffusion and broth dilution protocols.

**Bacterial strain**	***A. bicolor*/standard antibiotics**	**Antibacterial activity**	
		**ZOI (mm)**	**MIC (*μ*g/mL)**
*S. aureus*	100 mg/mL	12 ± 0.5	3125
	50 mg/mL	10.5 ± 1.2	
	25 mg/mL	8 ± 0.7	
	0 mg/mL	0	
	MP‐10 *μ*g	21 ± 1.6	1.56
	AMK‐30 *μ*g	22 ± 1.7	1.56

*K. pneumonia*	100 mg/mL	0	—
	50 mg/mL	0	
	25 mg/mL	0	
	0 mg/mL	0	
	MP‐10 *μ*g	25 ± 1.8	—
	AMK‐30 *μ*g	27 ± 1.2	—

*P. aeruginosa*	100 mg/mL	0	—
	50 mg/mL	0	
	25 mg/mL	0	
	0 mg/mL	0	
	MP‐10 *μ*g	18 ± 1.6	—
	AMK‐30 *μ*g	18 ± 0.6	—

*E. coli*	100 mg/mL	0	—
	50 mg/mL	0	
	25 mg/mL	0	
	0 mg/mL	0	
	MP‐10 *μ*g	21.3 ± 1.4	—
	AMK‐30 *μ*g	20 ± 0.8	—

*Note:* Zone of inhibition in mm expressed as mean ± SD—indicate not tested.

Abbreviations: AMK‐30 *μ*g, amikacin disc‐30 *μ*g (standard antibiotics); MP‐10 *μ*g, meropenem disc‐10 *μ*g (standard antibiotics); ZOI, zone of inhibition.

Several previous studies have demonstrated that phenolic acids and flavonoids bind via hydrogen bonds to disrupt the cell membrane [51], inhibit its synthesis, suppress enzyme synthesis, and reduce ATP formation, ultimately leading to bacterial death [49, 50, 52–54]. Specifically, quercetin causes the leakage of cellular electrolytes by significantly disrupting the cell walls and plasma membranes of *S. aureus* [54]. Similarly, chlorogenic acid has been demonstrated to inhibit the *S. aureus* proliferation by inhibiting efflux pumps and biofilm formation, destroying the membrane, and causing the release of intracellular constituents [55]. On the contrary, *A. bicolor* extracts did not show inhibitory activity against *E. coli* and *P. aeruginosa*. The resistance observed against *A. bicolor* extracts may be due to the limited penetration of their phytoconstituents caused by the presence of impermeable lipopolysaccharide in the outer cell membrane of gram‐negative bacterial strains [56].

### 3.8. Antidiabetic Activity

Carbohydrate‐rich meal, such as starch, is digested by the endogenous enzymes *α*‐amylase and *α*‐glucosidase into absorbable monosaccharides, causing rapid rise of blood glucose and leads to diabetic complication [31, 57]. Therefore, slowing the starch hydrolysis by inhibition of *α*‐amylase to decrease postprandial glycemia is considered as a potent target for novel antidiabetic drug development [58]. Previous scientific studies have gifted several potent *α*‐amylase and *α*‐glucosidase inhibitors to control blood sugar level. For instance, acarbose, miglitol, and voglibose are widely used worldwide to manage diabetes in clinical settings [58, 59]. Therefore, our research also concentrated on these well‐known targets to assess the antidiabetic potential of *A. bicolor* extract. In current enzymatic assay, *A. bicolor* extract showed a weak enzyme inhibitory activity, with an IC_50_ value (558.56 ± 0.76 *μ*g/mL), less potent than standard acarbose IC_50_ (41.24 ± 0.4 *μ*g/mL).

### 3.9. Molecular Docking

Molecular docking is a computational technique used in drug discovery and molecular biology to aid a deep understanding of the binding mechanisms between drugs and their target proteins, facilitating the design of novel therapeutic compounds [60]. In this study, the notable in vitro antibacterial activity of *A. bicolor* against *S. aureus* piqued our interest in exploring its underlying pharmacological mechanisms. Consequently, we performed in silico molecular docking analyses to investigate how the phytoconstituents of *A. bicolor* exert their antibacterial effects. We observed that the standard antibiotic meropenem, belonging to the PBP inhibitory class, was effective against *S. aureus*. Additionally, most of the natural compounds demonstrated antibacterial activity through the inhibition of PBP [22, 51]. Therefore, PBP was selected as the template for our in silico screening of antibacterial activity.

**Figure 13:**
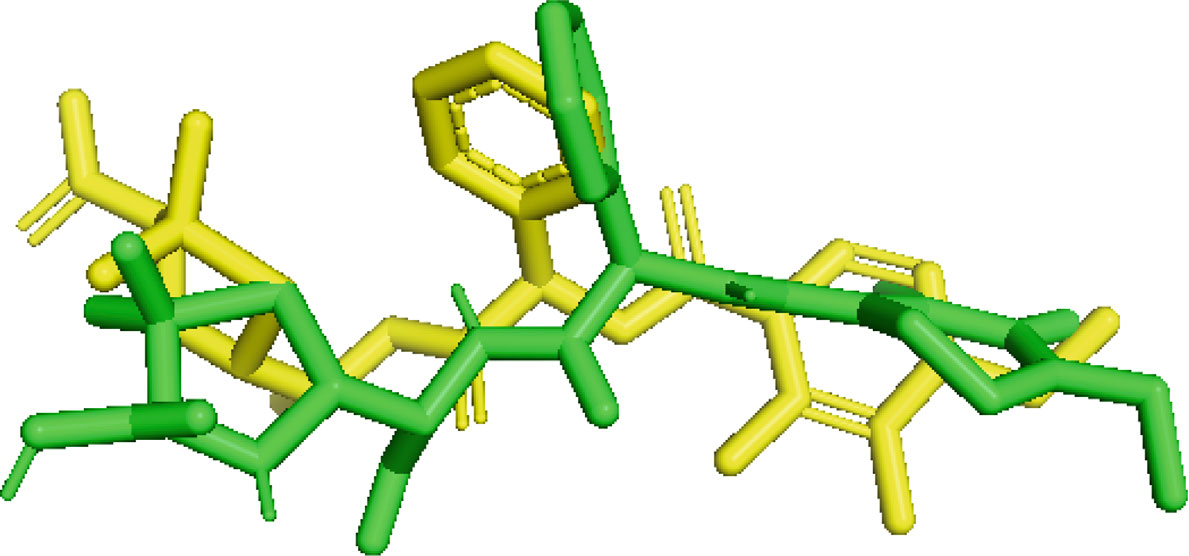
Validation of docking protocol with RMSD 1.181 Å via superposition of docked pose (green) and native pose (yellow) of co‐crystal ligand piperacillin bound with target protein PBP 2a.

PBP 2a is a membrane protein of *S. aureus* that catalyzes the critical transpeptidation cross‐linking polymerization reaction in bacterial peptidoglycans cell wall synthesis and is considered a potential target for novel antibacterial compounds [61, 62]. In predocking, an RMSD value of < 2 Å was obtained when the docked pose was superimposed with the native co‐crystal ligand (Figure 13), validating the docking protocol [24, 63]. Furthermore, the co‐crystalized piperacillin was mapped using BIOVIA Discovery Studio to identify the catalytic triad and active residues within the 3D crystal structure of host proteins; it discovered numerous active residues, including SER 403, LYS 406, TYR 446, SER 462, ASN 464, TYR 519, GLN 521, SER 598, GLY 599, THR 600, and MET 641 for the site‐specific docking studies [5].

In the docking study, all phytoconstituents revealed a prominent binding energy ranging from −6.3 to −7.1 kcal/mol, as compared with standard meropenem with a docking score of −7.4 kcal/mol. In BIOVIA Discovery Studio Visualizer, these phytoconstituents were observed to bind with almost all catalytic residues through conventional hydrogen bond, carbon hydrogen bond, pi–sigma interaction, pi–pi T‐shaped interaction, pi–donor hydrogen bond, pi–alkyl interaction, and pi–pi stacked molecular interactions, as displayed in Figure 14 and Table 4. Particularly, conventional hydrogen bonding was the dominant interaction in the context of quercetin 3‐*O*‐glucoside with prominent binding energy (−7.1 kcal/mol), indicating stable phytoconstituent–PBP interactions [64]; therefore, this supports the notable in vitro antibacterial inhibitory activity and provides scientific evidence validating the therapeutic potential of *A. bicolor* as an antibacterial crude drug. Moreover, our study entails further in vivo and cell line research for a comprehensive exploration of safety and efficacy.

Figure 143D and 2D molecular interactions of (a) chlorogenic acid, (b) quercetin 3‐*O*‐glucoside, (c) rhamnocitrin 3‐*O*‐glucoside, (d) kumatakenin, and (e) standard antibiotics meropenem within the catalytic pocket of PBP 2a.

**Figure:**
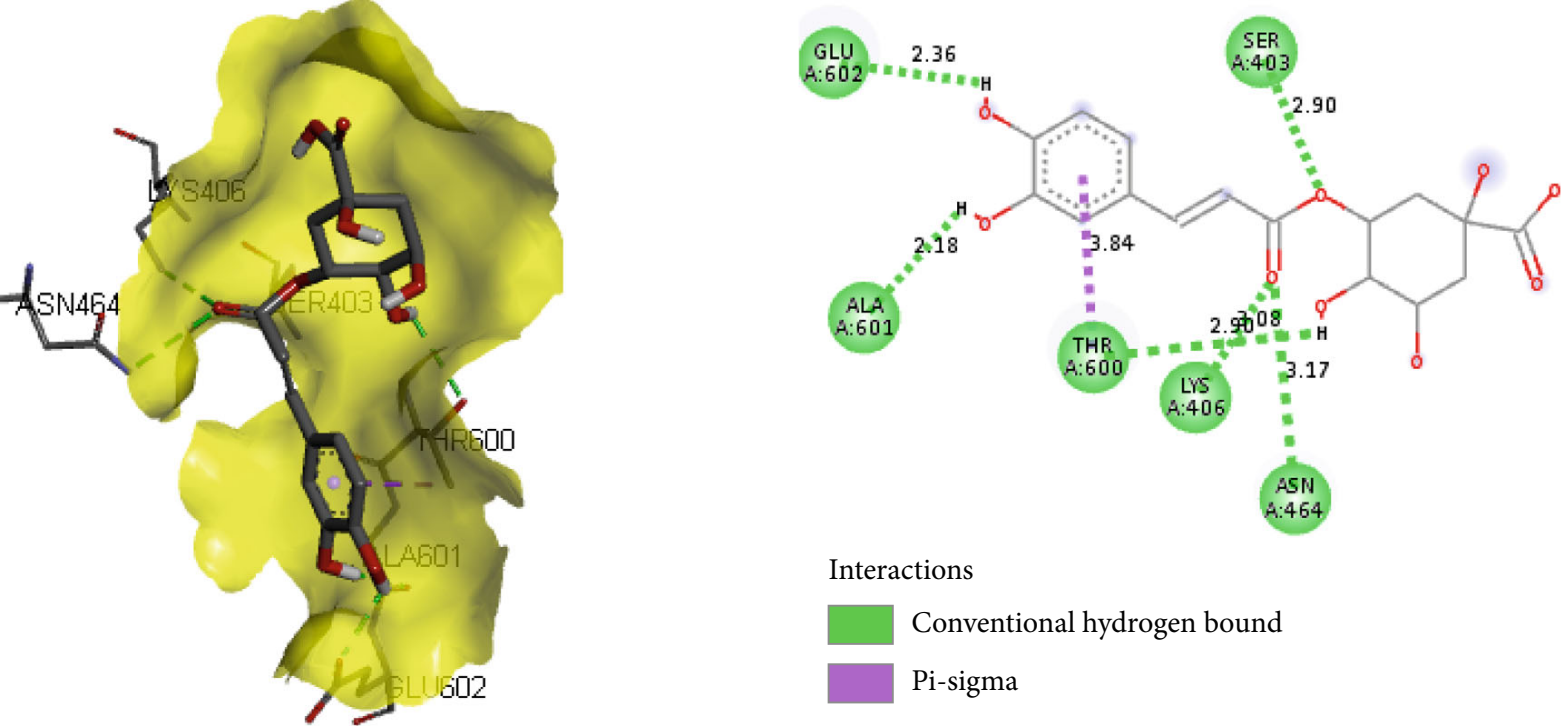
(a)

**Figure:**
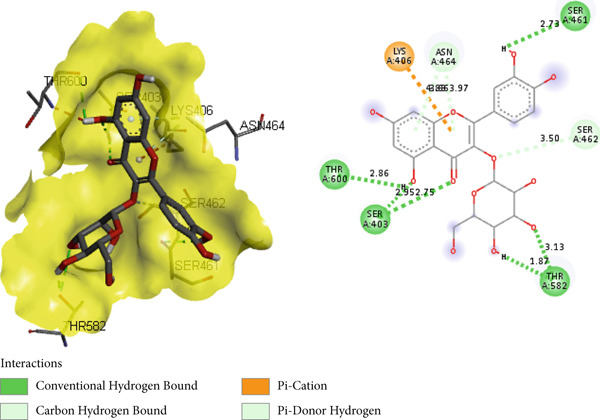
(b)

**Figure:**
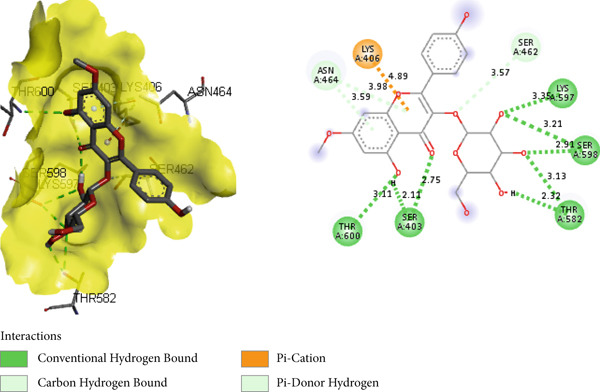
(c)

**Figure:**
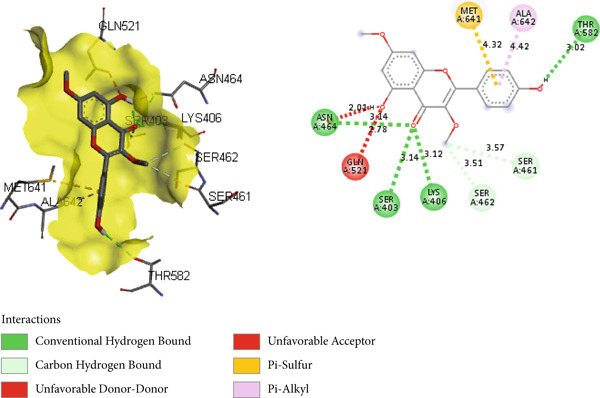
(d)

**Figure:**
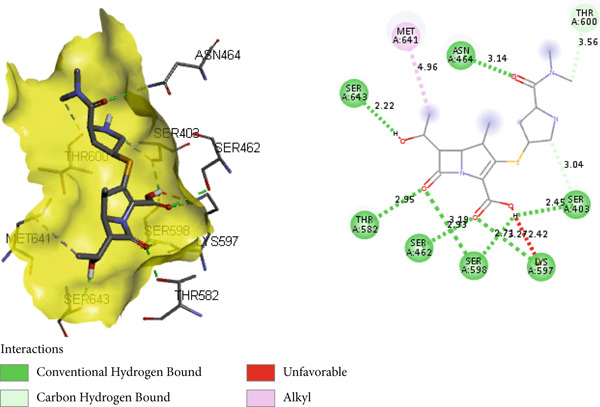
(e)

**Table 4:** Binding energy (kcal/mol) and molecular interaction of the *A. bicolor* phytochemicals with penicillin‐binding protein of *S. aureus*.

**Ligands**	***Staphylococcus aureus* PBP 2a (PDB ID: 6H5O)**	
	**Docking score (kcal/mol)**	**Active site residue interaction**
Chlorogenic acid	−6.9	SER 403, LYS 406, ASN 464, THR 600
Quercetin 3‐*O*‐glucoside	−7.1	SER 403, LYS 406, SER 462, ASN 464, THR 600
Rhamnocitrin 3‐*O*‐glucoside	−6.9	SER 403, LYS 406, SER 462, ASN 464, SER 598, THR 600
Kumatakenin	−6.3	SER 403, LYS 406, SER 462, ASN 464, GLN 521, THR 600, and MET 641
Meropenem	−7.4	SER 403, SER 462, ASN 464, TYR 519, SER 598, THR 600, and MET 641

### 3.10. Drug‐Likeliness

Estimation of pharmacokinetic properties, including absorption, distribution, metabolism, and excretion (ADME), is crucial for ensuring the effectiveness of bioactive phytoconstituents in the drug discovery and development process [1]. Approximately 40% of lead compound failures are attributed to ADMET issues [22]. Innovative in silico method, such as SwissADME, provides valuable and reliable tools for forecasting ADME profiles of drug candidates. These approaches offer notable advantages, including cost savings, rapid analysis, environmentally friendly predictions, and the potential to eliminate the need for animal testing [1, 21]. A fundamental principle in this context is Lipinski′s Rule of Five, which assists to determine pharmacokinetics of the natural compounds to be a potential drug candidate. To qualify as an ideal Lipinski′s drug candidate, lead compound must meet specific physiochemical criteria, including a molecular weight of **≤** 500 g/mol, no more than five hydrogen bond donors, no more than 10 hydrogen bond acceptors, 10 or fewer rotatable bonds, a topological polar surface area (TPSA) of ≤ 140 Å^2^, and lipophilicity (Log *P*_*o*/*w*_) less than five [65, 66]. Among the phytoconstituents analyzed, kumatakenin satisfied the Lipinski′s rule (Table 5), indicating excellent solubility, membrane permeability, and efficacy, making them strong candidates for further drug development [67].

**Table 5:** Drug‐likeness parameter of phytoconstituents from *A. bicolor* via the SwissADME server.

**S. N.**	**Compound**	**Mol. Wt. (g/mol)**	**Number of rotatable bonds**	**Number of H-bond acceptors**	**Number of H-bond donors**	**L** **o** **g** *P*_*o*/*w*_ **(MLOGP)**	**TPSA (Å)**	**Lipinski′s rule**
1	Chlorogenic acid	354.31	5	9	6	−1.05	164.75	No; 2 violation
2	Quercetin 3‐ *O*‐glucoside	464.38	4	12	8	−2.59	210.51	No; 2 violation
3	Rhamocitrin 3‐*O*‐glucoside	462.4	5	11	6	−1.89	164.75	No; 2 violation
4	Kumatakenin	314.29	3	6	2	0.47	89.13	Yes; 0 violation

## 4. Conclusions

This study offers a pioneering insight into the phytochemical profile and biological activities of *A. bicolor* extract, identifying key polyphenolic compounds such as chlorogenic acid, quercetin 3‐*O*‐glucoside, rhamnocitrin 3‐*O*‐glucoside, and kumatakenin through LC‐MS technique. The in vitro experiments demonstrated strong antioxidant activity, particularly in scavenging DPPH free radicals, along with moderate antibacterial effects against *S. aureus*, supporting its traditional medicinal values. Complementary in silico molecular docking studies further indicated the potential interactions between these phytoconstituents and active site residues of the penicillin‐binding protein of *S. aureus*, reinforcing the in vitro bioactivity findings and suggesting antibacterial therapeutic prospects for traditional uses. However, the study faces limitations, including the need for advanced analytical techniques, detailed isolation of bioactive compounds, in vivo validation, and safety evaluations. As a result, these findings lay the groundwork for further in‐depth investigations aimed at discovering and developing novel antioxidant and antibacterial agents from *A. bicolor*.

## Disclosure

All authors have agreed to the publication of this article.

## Conflicts of Interest

The authors declare no conflicts of interest.

## Author Contributions

Rekha Bhandari, Shailendra Kumar Sharma, Peru Kumari Bishwakarma, Sadikshya Sapkota, and Ram Kishor Yadav participated in the conceptualization, investigations, methodology, data curation, original draft writing, and review and editing; Khem Raj Joshi participated in methodology, investigation, conceptualization, supervision, and validation; Harish Babu P.C., Sandesh Poudel, and Sajan L. Shyaula participated in methodology, investigation, formal analysis, and draft review and editing.

## Funding

No funding was received for this manuscript.

## Data Availability

The data that support the findings of this study are included in the manuscript.

## References

[1] Yadav R. K. , Panta S. , Sindhu K. C. , Jha P. K. , Poudel S. , Upadhyaya S. R. , Subedi K. , Baral S. , Bhandari R. , and Pandey B. , LC-MS Analysis and Evaluation of Antioxidant and Antibacterial Activities of *Falconeria insignis* Royle From Nepal: An In Vitro/In Silico Approach, Natural Product Communications. (2025) 20, no. 5, 10.1177/1934578X251340467.

[2] Khan A. , Sajjad M. , and Ahmad I. , Khan M. S. A. , Ahmad I. , and Debprasad B. T. , Chapter 1—Herbal Medicine: Current Trends and Future Prospects, New Look to Phytomedicine Chattopadhyay, 2019, Academic Press, 3–13, 10.1016/B978-0-12-814619-4.00001-X.

[3] Swaraz A. M. , Sultana F. , Bari M. W. , Ahmed K. S. , Hasan M. , Islam M. M. , Islam M. A. , Satter M. A. , Hossain M. H. , Islam M. S. , Khan M. I. , and Raihan M. O. , Phytochemical Profiling of *Blumea laciniata* (Roxb.) DC. and Its Phytopharmaceutical Potential Against Diabetic, Obesity, and Alzheimer′s, Biomedicine & Pharmacotherapy. (2021) 141, 111859, 10.1016/j.biopha.2021.111859, 34246953. 34246953

[4] Ahn K. , The Worldwide Trend of Using Botanical Drugs and Strategies for Developing Global Drugs, BMB Reports. (2017) 50, no. 3, 111–116, 10.5483/BMBRep.2017.50.3.221, 2-s2.0-85016631096, 27998396. 27998396 PMC5422022

[5] Yadav R. K. , Bhandari R. , Harish Babu P. C. , Jha P. K. , Pandey B. , Kc S. , Upadhaya S. R. , Panta S. , Shyaula S. L. , and Joshi K. R. , LC-MS Analysis and Antioxidant, Antibacterial, and Antidiabetic Activity of Jumli Marshi Rice From Nepal: An In Vitro and In Silico Investigation to Validate Their Potential as a Functional Food, PLoS One. (2025) 20, no. 3, e0319338, 10.1371/journal.pone.0319338. 40063879 PMC11893129

[6] Chen M. , He X. , Sun H. , Sun Y. , Li L. , Zhu J. , Xia G. , Guo X. , and Zang H. , Phytochemical Analysis, UPLC-ESI-Orbitrap-MS Analysis, Biological Activity, and Toxicity of Extracts From *Tripleurospermum limosum* (Maxim.) Pobed, Arabian Journal of Chemistry. (2022) 15, no. 5, 103797, 10.1016/j.arabjc.2022.103797.

[7] Mandal F. , Sindhu K. C. , Yadav S. R. , Kumar S. , Mishra P. K. , Shrestha S. , and Khan M. B. , Association Between Thyroid Function and Oxidative Stress Markers in Overt and Subclinical Hypothyroid Patients: Observational Cross-Sectional Study in Nepal, Palestinian Medical and Pharmaceutical Journal. (2025) 10, no. 2, 157–164, 10.59049/2790-0231.10.2.2311.

[8] Rawal P. , Pandey B. , Yadav R. K. , and Panta S. , Antioxidant, Alpha-Amylase Inhibitory and Hypoglycemic Activity of *Smallanthus sonchifolius* Leaves From Nepal: An Integrated In Vitro, In Vivo, and In Silico Approach, Food Science & Nutrition. (2025) 13, no. 1, e4672, 10.1002/fsn3.4672, 39803296. 39803296 PMC11717019

[9] Ita B. N. and Eduok S. I. , In Vitro Antioxidant and Antifungal Activities of *Rhizophora racemosa* G.F.W. Mey. Stem Bark Extracts, Scientific African. (2022) 15, e01091, 10.1016/j.sciaf.2021.e01091.

[10] Truong D. H. , Nguyen D. H. , Ta N. T. A. , Bui A. V. , Do T. H. , and Nguyen H. C. , Evaluation of the Use of Different Solvents for Phytochemical Constituents, Antioxidants, and In Vitro Anti-Inflammatory Activities of *Severinia buxifolia* , Journal of Food Quality. (2019) 2019, 10.1155/2019/8178294, 2-s2.0-85062349776, 8178294.

[11] Sharaf M. H. , Abdelaziz A. M. , Kalaba M. H. , Radwan A. A. , and Hashem A. H. , Antimicrobial, Antioxidant, Cytotoxic Activities and Phytochemical Analysis of Fungal Endophytes Isolated from *Ocimum basilicum* , Applied Biochemistry and Biotechnology. (2022) 194, no. 3, 1271–1289, 10.1007/s12010-021-03702-w, 34661866. 34661866

[12] Angelini P. , Plant-Derived Antimicrobials and Their Crucial Role in Combating Antimicrobial Resistance, Antibiotics. (2024) 13, no. 8, 10.3390/antibiotics13080746, 39200046. PMC11350763 39200046

[13] Li Z. , He Q. , Feifei X. , Yin X. , Guan Z. , Song J. , He Z. , Yang X. , and Situ C. , Exploring the Antibacterial Potential and Underlying Mechanisms of *Prunella vulgaris* L. on Methicillin-Resistant *Staphylococcus aureus* , Foods. (2024) 13, no. 5, 10.3390/foods13050660. PMC10931123 38472772

[14] Pandey B. , Yadav R. K. , Subedi L. , Sapkota B. , Baral M. , Jha P. K. , KC S. , and Panta S. , Phytochemical Investigation and Antioxidant Activity ofMillettia extensaAgainst Mushroom Tyrosinase Enzyme: Molecular Insight Into Skin Care Products, Natural Product Communications. (2025) 20, no. 1, 10.1177/1934578X251318518.

[15] Sindhu K. C. , Kaundinnyayana A. , Jha P. K. , Poudel S. , Tiwari R. , Yadav R. K. , Sistu K. C. , and Subedi K. , Phytochemical Constituents and In-Vitro Antioxidant Activity of *Aleuritopteris bicolor* Leaves, Crinum amoenum Bulbs , and Drynaria coronans Rhizomes of Nepal, Jordan Journal of Pharmaceutical Sciences. (2025) 18, no. 2, 555–565, 10.35516/jjps.v18i2.2691.

[16] Ojha R. and Devkota H. , Edible and Medicinal Pteridophytes of Nepal: A Review, Ethnobotany Research and Applications. (2021) 22, 10.32859/era.22.16.1-16.

[17] Adhikari M. , Thapa R. , Kunwar R. M. , Devkota H. P. , and Poudel P. , Ethnomedicinal Uses of Plant Resources in the Machhapuchchhre Rural Municipality of Kaski District, Nepal, Medicine. (2019) 6, no. 2, 10.3390/medicines6020069. PMC6630641 31234605

[18] Tiwari R. , Baral R. , Parajuli N. , Shrestha R. , Pahari A. , and Gurung S. , Phytochemical Screening , Free Radical Scavenging and In-Vitro Anti-Bacterial Activity of Ethanolic Extracts of Selected Medicinal Plants of Nepal and Effort Towards Formulation of Antibacterial Cream From the Extracts, International Journal of Herbal Medicine. (2021) 9, no. 3, 39–47.

[19] Yadav R. K. , Shrestha P. , Timilsina K. , Dhakal A. , Poudel S. , Sindhu K. C. , Jha P. K. , Paneru S. , Bhandari R. , and Joshi K. R. , Antioxidant, Antibacterial Activity, In Silico Molecular Docking, and ADME-Toxicity Study of Lactone From Rhizome of *Angiopteris helferiana* , Journal of Chemistry. (2024) 2024, 5623028, 10.1155/2024/5623028.

[20] Chen T. , Shuang F. F. , Qing Yue F. , Yu Xiong J. , Zong C. M. , Zhao W. G. , Zhang D. Y. , Yao X. H. , and Cao F. L. , Evaluation of the Chemical Composition and Antioxidant Activity of Mulberry (*Morus alba* L.) Fruits From Different Varieties in China, Molecules. (2022) 27, no. 9, 10.3390/molecules27092688. PMC9102544 35566039

[21] Shrestha M. , Sindhu K. C. , Sah B. , Jha P. K. , Khaitu S. , Pandey B. , Yadav R. K. , Gautam A. , and Yadav B. , Hydroethanolic Leaf Extract of *Murraya koenigii*: Phytochemical Constituents and Biological Evaluation of Its Toxicity and Antipyretic Activity in Wistar Albino Rats, Jordan Journal of Pharmaceutical Sciences. (2024) 17, no. 4, 811–817, 10.35516/jjps.v17i4.2532.

[22] Yadav R. K. , Dhakal A. , Timilsina K. , Shrestha P. , Poudel S. , Kc S. , Jha P. K. , Bhandari R. , and Joshi K. R. , Antioxidant and Antibacterial Activities Evaluation, Phytochemical Characterisation of Rhizome From *Angiopteris helferiana* and Barks From *Saurauia fasciculata* in Nepal, Scientific World Journal. (2024) 2024, 1119165, 10.1155/2024/1119165. 38898935 PMC11186685

[23] Hemeg H. A. , Moussa I. M. , Ibrahim S. , Dawoud T. M. , Alhaji J. H. , Mubarak A. S. , Kabli S. A. , Alsubki R. A. , Tawfik A. M. , and Marouf S. A. , Antimicrobial Effect of Different Herbal Plant Extracts Against Different Microbial Population, Saudi Journal of Biological Sciences. (2020) 27, no. 12, 3221–3227, 10.1016/j.sjbs.2020.08.015, 33304127. 33304127 PMC7714981

[24] Warren G. L. , Andrews C. W. , Capelli A.-M. , Clarke B. , LaLonde J. , Lambert M. H. , Lindvall M. , Nevins N. , Semus S. F. , Senger S. , Tedesco G. , Wall I. D. , Woolven J. M. , Peishoff C. E. , and Head M. S. , A Critical Assessment of Docking Programs and Scoring Functions, Journal of Medicinal Chemistry. (2006) 49, no. 20, 5912–5931, 10.1021/jm050362n, 2-s2.0-33749260698. 17004707

[25] Joshi K. R. , Devkota H. P. , Watanabe T. , and Yahara S. , Phenolic Compounds From the Flowers of Nepalese Medicinal Plant Aconogonon Molle and Their DPPH Free Radical-Scavenging Activities, Natural Product Research. (2014) 28, no. 23, 2208–2210, 10.1080/14786419.2014.915829, 2-s2.0-84908568234, 24825068. 24825068

[26] Joshi K. R. , Devkota H. P. , Watanabe T. , and Yahara S. , Thotneosides A, B and C: Potent Antioxidants From Nepalese Crude Drug, Leaves of Aconogonon Molle, Chemical and Pharmaceutical Bulletin. (2014) 62, no. 2, 191–195, 10.1248/cpb.c13-00748, 2-s2.0-84894265284, 24292866. 24292866

[27] Joshi K. R. , Devkota H. P. , and Yahara S. , Chemical Analysis of Flowers ofBombax ceibafrom Nepal, Natural Product Communications. (2013) 8, no. 5, 10.1177/1934578X1300800508.

[28] Chaves J. O. , De Souza M. C. , Capelasso L. , Forster-carneiro T. , Vázquez-espinosa M. , González-de-peredo A. V. , and Barbero G. F. , Extraction of Flavonoids From Natural Sources Using Modern Techniques, Frontiers in Chemistry. (2020) 8, 10.3389/fchem.2020.507887, 33102442. PMC7546908 33102442

[29] Weldegebrieal G. K. , Synthesis Method , Antibacterial and Photocatalytic Activity of ZnO Nanoparticles for Azo Dyes in Wastewater Treatment: A Review, Inorganic Chemistry Communications. (2020) 120, 108140, 10.1016/j.inoche.2020.108140.

[30] Jha P. K. , Sindhu K. C. , Yadav R. K. , Pandey B. , Paudel S. , Khadka R. , Subedi K. , and Panta S. , Exploring the Therapeutic Promise of Drynaria coronans: Phytochemical Analysis, Antioxidant Capacity, *α*-Amylase Inhibition with Safety Assessment, Journal of Medicinal Natural Products. (2025) 2, no. 1, 10.53941/jmnp.2025.100004, 100004.

[31] Jha P. K. , Pandey B. , Yadav R. K. , Sindhu K. C. , Poudel S. , and Panta S. , Anti-Inflammatory, Analgesic, Antioxidant, and Alpha-Amylase Inhibitory Effects of the Hydroethanolic Leaf Extract of *Aleuritopteris bicolor* (Roxb.) Fraser-Jenk, PLoS One. (2025) 20, no. 6, e0326808, 10.1371/journal.pone.0326808. 40549787 PMC12184904

[32] Devkota H. P. , Adhikari-Devkota A. , Takano A. , Yahara S. , and Basnet P. , Journal of Nepal Pharmaceutical Association, Journal of Nepal Pharmaceutical Association. (2017) 28, 1–11.

[33] Pitt J. J. , Principles and Applications of Liquid Chromatography-Mass Spectrometry in Clinical Biochemistry, Clinical Biochemist Reviews. (2009) 30, no. 1, 19–34, 19224008. 19224008 PMC2643089

[34] Kruve A. and Kaupmees K. , Adduct Formation in ESI/MS by Mobile Phase Additives, Journal of the American Society for Mass Spectrometry. (2017) 28, no. 5, 887–894, 10.1007/s13361-017-1626-y, 2-s2.0-85017462997, 28299714. 28299714

[35] Zhu J. and Cole R. B. , Formation and Decompositions of Chloride Adduct Ions, [M + Cl]−, in Negative Ion Electrospray Ionization Mass Spectrometry, Journal of the American Society for Mass Spectrometry. (2000) 11, no. 11, 932–941, 10.1016/S1044-0305(00)00164-1, 2-s2.0-0034488256, 11073256. 11073256

[36] Lamichhane R. , Kim S.-g. , Poudel A. , Sharma D. , Lee H. , Poudel P. , Pandeya P. R. , and Jung H.-j. , Identification of Flavonoids fromCheilanthes albomarginataClarke and Their Simultaneous Determination and Quantification by UPLC/DAD Method, Journal of Liquid Chromatography & Related Technologies. (2015) 38, no. 19, 1713–1721, 10.1080/10826076.2015.1091011, 2-s2.0-84948972401.

[37] Pearson J. L. , Lee S. , Suresh H. , Low M. , Nang M. , Singh S. , Lamin F. , Kazzem M. , Sullivan S. , and Khoo C. S. , The Liquid Chromatographic Determination of Chlorogenic and Caffeic Acids in Xu Duan (*Dipsacus asperoides*) Raw Herb, International Scholarly Research Notices. (2014) 2014, 10.1155/2014/968314, 968314.

[38] Yonathan M. , Asres K. , Assefa A. , and Bucar F. , In Vivo Anti-Inflammatory and Anti-Nociceptive Activities of *Cheilanthes farinosa* , Journal of Ethnopharmacology. (2006) 108, no. 3, 462–470, 10.1016/j.jep.2006.06.006, 2-s2.0-33750627695. 16876348

[39] Verma D. D. , Antioxidative Properties of Flavonoids from Cheilanthes Anceps Swartz, Journal of American Science, 2010, 6, no. 5.

[40] Bai Y. , Zheng Y. , Pang W. , Peng W. , Hao W. , Yao H. , Li P. , Deng W. , Cheng J. , and Weiwei S. , Identification and Comparison of Constituents of Aurantii Fructus and Aurantii Fructus Immaturus by UFLC-DAD-Triple TOF-MS/MS, Molecules. (2018) 23, no. 4, 10.3390/molecules23040803, 2-s2.0-85044817251, 29601542. PMC6017871 29601542

[41] Fabre N. , Rustan I. , de Hoffmann E. , and Quetin-Leclercq J. , Determination of Flavone, Flavonol, and Flavanone Aglycones by Negative Ion Liquid Chromatography Electrospray Ion Trap Mass Spectrometry, Journal of the American Society for Mass Spectrometry. (2001) 12, no. 6, 707–715, 10.1016/S1044-0305(01)00226-4, 2-s2.0-0035567362, 11401161. 11401161

[42] Prasathkumar M. , Raja K. , Vasanth K. , Khusro A. , Sadhasivam S. , Sahibzada M. U. , Gawwad M. R. , Al Farraj D. A. , and Elshikh M. S. , Phytochemical Screening and In Vitro Antibacterial, Antioxidant, Anti-Inflammatory, Anti-Diabetic, and Wound Healing Attributes of *Senna auriculata* (L.) Roxb. Leaves, Arabian Journal of Chemistry. (2021) 14, no. 9, 103345, 10.1016/j.arabjc.2021.103345.

[43] Ihsanpuro S. I. , Gunawan S. , Ibrahim R. , and Aparamarta H. W. , Extract With High 1,1-Diphenyl-2-Picrylhydrazyl (DPPH) Inhibitory Capability From Pericarp and Seed of Mangosteen (*Garcinia mangostana* L.) Using Microwave-Assisted Extraction (MAE) Two-Phase Solvent Technique, Arabian Journal of Chemistry. (2022) 15, no. 12, 104310, 10.1016/j.arabjc.2022.104310.

[44] Amin M. U. , Khurram M. , Khattak B. , and Khan J. , Antibiotic Additive and Synergistic Action of Rutin, Morin and Quercetin Against Methicillin Resistant *Staphylococcus aureus* , BMC Complementary and Alternative Medicine. (2015) 15, no. 1, 10.1186/s12906-015-0580-0, 2-s2.0-84925039459, 25879586. PMC4364681 25879586

[45] Betts J. W. , Sharili A. S. , Phee L. M. , and Wareham D. W. , In Vitro Activity of Epigallocatechin Gallate and Quercetin Alone and in Combination Versus Clinical Isolates of Methicillin-Resistant *Staphylococcus aureus* , Journal of Natural Products. (2015) 78, no. 8, 2145–2148, 10.1021/acs.jnatprod.5b00471, 2-s2.0-84940547044, 26267658. 26267658

[46] Hirai I. , Okuno M. , Katsuma R. , Arita N. , Tachibana M. , and Yamamoto Y. , Characterisation of Anti-*Staphylococcus aureus* Activity of Quercetin, International Journal of Food Science and Technology. (2010) 45, no. 6, 1250–1254, 10.1111/j.1365-2621.2010.02267.x, 2-s2.0-77955562995.

[47] Ivanov M. , Novović K. , Malešević M. , Dinić M. , Stojković D. , Jovčić B. , and Soković M. , Polyphenols as Inhibitors of Antibiotic Resistant Bacteria—Mechanisms Underlying Rutin Interference With Bacterial Virulence, Pharmaceuticals. (2022) 15, no. 3, 10.3390/ph15030385, 35337182. PMC8952364 35337182

[48] Jaisinghani R. N. , Antibacterial Properties of Quercetin, Microbiology Research. (2017) 8, no. 1, 10.4081/mr.2017.6877.

[49] Keyvani-Ghamsari S. , Rahimi M. , and Khorsandi K. , An Update on the Potential Mechanism of Gallic Acid as an Antibacterial and Anticancer Agent, Food Science and Nutrition. (2023) 11, no. 10, 5856–5872, 10.1002/fsn3.3615, 37823155. 37823155 PMC10563697

[50] Pinho E. , Ferreira I. C. F. R. , Barros L. , Carvalho A. M. , Soares G. , and Henriques M. , Antibacterial Potential of Northeastern Portugal Wild Plant Extracts and Respective Phenolic Compounds, BioMed Research International. (2014) 2014, 10.1155/2014/814590, 2-s2.0-84900024999, 814590. 24804249 PMC3997077

[51] Calvo L. G. , Castillo A. , Villarino R.-A. , Rama J. L. R. , Abril A. G. , and de Miguel T. , Study of the Antibacterial Activity of Rich Polyphenolic Extracts Obtained from Cytisus scoparius Against Foodborne Pathogens, Antibiotics. (2023) 12, no. 11, 10.3390/antibiotics12111645, 37998847. PMC10669525 37998847

[52] Miklasińska-Majdanik M. , Kępa M. , Wojtyczka R. D. , Idzik D. , and Wąsik T. J. , Phenolic Compounds Diminish Antibiotic Resistance of Staphylococcus aureus Clinical Strains, International Journal of Environmental Research and Public Health. (2018) 15, no. 10, 10.3390/ijerph15102321, 2-s2.0-85055648934, 30360435. PMC6211117 30360435

[53] Mostafa A. A. , Al-Askar A. A. , Almaary K. S. , Dawoud T. M. , Sholkamy E. N. , and Bakri M. M. , Antimicrobial Activity of Some Plant Extracts Against Bacterial Strains Causing Food Poisoning Diseases, Saudi Journal of Biological Sciences. (2018) 25, no. 2, 361–366, 10.1016/j.sjbs.2017.02.004, 2-s2.0-85015752349, 29472791. 29472791 PMC5815983

[54] Anh N. T. L. and Bhattacharya D. , Antimicrobial Activity of Quercetin: An Approach to Its Mechanistic Principle, Molecules. (2022) 27, no. 8, 10.3390/molecules27082494. PMC9029217 35458691

[55] Sheikhy M. , Karbasizade V. , Ghanadian M. , Fazeli H. , Basireddy S. R. , and Alsultan A. , Evaluation of Chlorogenic Acid and Carnosol for Anti-Efflux Pump and Anti-Biofilm Activities against Extensively Drug-Resistant Strains of *Staphylococcus aureus* and *Pseudomonas aeruginosa* , Microbiology Spectrum. (2024) 12, no. 9, e0393423, 10.1128/spectrum.03934-23, 39046262. 39046262 PMC11370622

[56] Biswas B. , Rogers K. , McLaughlin F. , Daniels D. , and Yadav A. , Antimicrobial Activities of Leaf Extracts of Guava (*Psidium guajava* L.) on Two Gram-Negative and Gram-Positive Bacteria, International Journal of Microbiology. (2013) 2013, 10.1155/2013/746165, 2-s2.0-84887455593, 746165. 24223039 PMC3817707

[57] Nasir A. , Khan M. , Rehman Z. , Khalil A. A. K. , Farman S. , Begum N. , Irfan M. , Sajjad W. , and Parveen Z. , Evaluation of Alpha-Amylase Inhibitory, Antioxidant, and Antimicrobial Potential and Phytochemical Contents of *Polygonum hydropiper* L., Plants. (2020) 9, no. 7, 10.3390/plants9070852, 32640649. PMC7412011 32640649

[58] Williams L. K. , Zhang X. , Caner S. , Tysoe C. , Nguyen N. T. , Wicki J. , Williams D. E. , Coleman J. , McNeill J. H. , Yuen V. , Andersen R. J. , Withers S. G. , and Brayer G. D. , The Amylase Inhibitor Montbretin A Reveals a New Glycosidase Inhibition Motif, Nature Chemical Biology. (2015) 11, no. 9, 691–696, 10.1038/nchembio.1865, 2-s2.0-84939568633, 26214255. 26214255

[59] Oboh G. , Agunloye O. M. , Adefegha S. A. , Akinyemi A. J. , and Ademiluyi A. O. , Caffeic and Chlorogenic Acids Inhibit Key Enzymes Linked to Type 2 Diabetes (In Vitro): A Comparative Study, Journal of Basic and Clinical Physiology and Pharmacology. (2015) 26, no. 2, 165–170, 10.1515/jbcpp-2013-0141, 2-s2.0-84937937489, 24825096. 24825096

[60] Dilshad R. , Khan K.-u.-R. , Dilshad R. , Ahmad S. , Rao H. , Khurshid U. , Ahmad S. , Ahmad M. , Abid H. M. U. , Zaman M. K. , Nisar R. , Khaliq S. , and Ghalloo B. A. , Comprehensive Chemical Profiling With UHPLC-MS, In-Vitro, In-Silico, and In-Vivo Antidiabetic Potential of Typha domingensis Pers; a Novel Source of Bioactive Compounds, South African Journal of Botany. (2024) 171, 185–198, 10.1016/j.sajb.2024.06.007.

[61] Ambade S. S. , Gupta V. K. , Bhole R. P. , Khedekar P. B. , and Chikhale R. V. , A Review on Five and Six-Membered Heterocyclic Compounds Targeting the Penicillin-Binding Protein 2 (PBP2A) of Methicillin-Resistant Staphylococcus Aureus (MRSA), Molecules. (2023) 28, no. 20, 10.3390/molecules28207008, 37894491. PMC10609489 37894491

[62] Aribisala J. O. and Sabiu S. , Cheminformatics Identification of Phenolics as Modulators of Penicillin-Binding Protein 2a of Staphylococcus Aureus: A Structure–Activity-Relationship-Based Study, Pharmaceutics. (2022) 14, no. 9, 10.3390/pharmaceutics14091818, 36145565. PMC9503099 36145565

[63] Pandey B. , Thapa S. , Biradar M. S. , Singh B. , Ghale J. B. , Kharel P. , Jha P. K. , Yadav R. K. , Dawadi S. , and Poojashree V. , LC-MS Profiling and Cytotoxic Activity of *Angiopteris helferiana* Against HepG2 Cell Line: Molecular Insight to Investigate Anticancer Agent, PLoS One. (2024) 19, no. 12, e0309797, 10.1371/journal.pone.0309797, 39739862. 39739862 PMC11687663

[64] Abdullahi S. H. , Uzairu A. , Shallangwa G. A. , Uba S. , and Umar A. B. , Molecular Docking, ADMET and Pharmacokinetic Properties Predictions of Some Di-Aryl Pyridinamine Derivatives as Estrogen Receptor (Er+) Kinase Inhibitors, Egyptian Journal of Basic and Applied Sciences. (2022) 9, no. 1, 180–204, 10.1080/2314808X.2022.2050115.

[65] Ali M. , Hassan M. , Ansari S. A. , Alkahtani H. M. , Al-Rasheed L. S. , and Ansari S. A. , Quercetin and Kaempferol as Multi-Targeting Antidiabetic Agents Against Mouse Model of Chemically Induced Type 2 Diabetes, Pharmaceuticals. (2024) 17, no. 6, 10.3390/ph17060757, 38931424. PMC11206732 38931424

[66] Anusionwu C. G. , Fonkui T. Y. , Oselusi S. O. , Egieyeh S. A. , Aderibigbe B. A. , and Mbianda X. Y. , Ferrocene-Bisphosphonates Hybrid Drug Molecules: In Vitro Antibacterial and Antifungal, In Silico ADME, Drug-Likeness, and Molecular Docking Studies, Results in Chemistry. (2024) 7, 101278, 10.1016/j.rechem.2023.101278.

[67] Morak-Młodawska B. , Pluta K. , and Jeleń M. , Evaluation of the Lipophilicity of New Anticancer 1,2,3-Triazole-Dipyridothiazine Hybrids Using RP TLC and Different Computational Methods, Processes. (2020) 8, no. 7, 10.3390/pr8070858.

